# A dynamic evolutionary and functional landscape of plant phased small interfering RNAs

**DOI:** 10.1186/s12915-015-0142-4

**Published:** 2015-05-16

**Authors:** Yi Zheng, Ying Wang, Jian Wu, Biao Ding, Zhangjun Fei

**Affiliations:** 1 Boyce Thompson Institute for Plant Research, Cornell University, Ithaca, NY 14853 USA; 2 Department of Molecular Genetics, The Ohio State University, Columbus, OH 43210 USA; 3 The Center for RNA Biology, The Ohio State University, Columbus, OH 43210 USA; 4 Molecular, Cellular and Developmental Biology Program, The Ohio State University, Columbus, OH 43210 USA; 5 USDA Robert W. Holley Center for Agriculture and Health, Tower Road, Ithaca, NY 14853 USA

**Keywords:** Evolution, Function, Plant phasiRNAs, Protein-coding genes

## Abstract

**Background:**

Secondary, phased small interfering RNAs (phasiRNAs) derived from protein-coding or noncoding loci (*PHAS*) are emerging as a new type of regulators of gene expression in plants. However, the evolution and function of these novel siRNAs in plant species remain largely unexplored.

**Results:**

We systematically analyzed *PHAS* loci in 23 plant species covering major phylogenetic groups spanning alga, moss, gymnosperm, basal angiosperm, monocot, and dicot. We identified over 3,300 *PHAS* loci, among which ~1,600 were protein-coding genes. Most of these *PHAS* loci were novel and clade- or species-specific and showed distinct expression patterns in association with particular development stages, viral infection, or abiotic stresses. Unexpectedly, numerous *PHAS* loci produced phasiRNAs from introns or exon–intron junction regions. Our comprehensive analysis suggests that phasiRNAs predominantly regulate protein-coding genes from which they are derived and genes from the same families of the phasiRNA-deriving genes, in contrast to the dominant *trans*-regulatory mode of miRNAs. The stochastic occurrence of many *PHAS* loci in the plant kingdom suggests their young evolutionary origins.

**Conclusions:**

Our study discovered an unprecedented diversity of protein-coding genes that produce phasiRNAs in a wide variety of plants, and set a kingdom-wide foundation for investigating the novel roles of phasiRNAs in shaping phenotype diversities of plants.

**Electronic supplementary material:**

The online version of this article (doi:10.1186/s12915-015-0142-4) contains supplementary material, which is available to authorized users.

## Background

The workings of combinatorial genetic regulatory networks control how an organism grows and develops as well as responds to biotic and abiotic stresses. Distinct regulatory networks emerged during evolution likely have contributed to the diversification of biological phenotypes. Investigations over the last decade from many different organisms have uncovered the novel regulatory roles of numerous small, noncoding RNAs. These include microRNAs (miRNAs) and short interfering RNAs (siRNAs) that exert epigenetic regulation of gene expression at the transcriptional, posttranscriptional, and translational levels [1–3]. These discoveries have led to RNA-based new paradigms of genetic regulatory networks that control various biological processes including the emergence of biological diversity [4–6].

In plants, miRNAs can trigger the generation of secondary, phased siRNAs (phasiRNAs) from protein-coding or intergenic loci (*PHAS*) [7]. Some phased siRNAs can *trans*-regulate the expression of target genes, and are called *trans*-acting siRNAs (tasiRNAs or TAS) [8]. Early studies with *Arabidopsis thaliana* provided genetic evidence supporting that some tasiRNAs regulate gene expression critical to plant development [9, 10], and it has been shown that a deeply conserved TAS3 pathway is pivotal in developmental processes from moss to higher plants [11–14]. More recent studies showed the generation of phasiRNAs from mRNAs that encode many nucleotide binding site (NBS) and leucine-rich repeat (LRR) disease resistance proteins in legumes [15, 16] and *Solanaceae* [17, 18]. Some disease resistance gene-derived phasiRNAs have been shown to down-regulate the expression of genes in the same family [19]. Besides NBS-LRR disease resistance proteins, many other types of protein-coding genes have been identified as *PHAS* loci in plants, such as MYB transcription factors [16, 20, 21], pentatricopeptide repeat (PPR) genes [16, 20, 22, 23], transporter inhibitor response (TIR)/auxin F-box genes (AFB) [15, 22, 24, 25], and calcium ATPase transporters [23, 26].

**Fig. 1:**
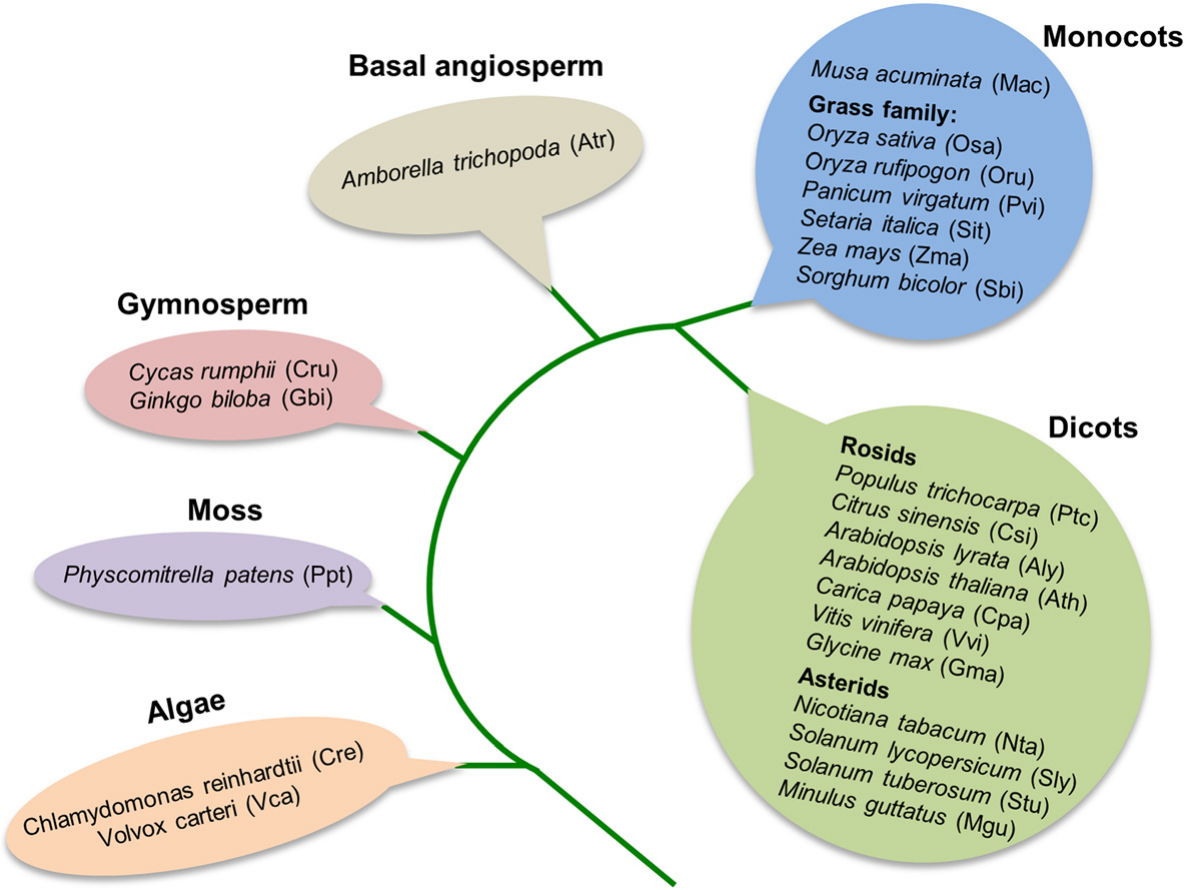
Plant species investigated in this study. These species belong to major groups with well-established phylogeny. Three-letter abbreviations of these species, shown in brackets, were used in the present study

These findings suggest potentially broad roles of phasiRNAs in plant evolution and function, but many fundamental questions remain outstanding: (1) How widespread are *PHAS* loci in the plant kingdom? (2) How many types of protein-coding genes can function as *PHAS* loci in the plant kingdom? (3) How conserved or unique are these *PHAS* loci in different plants? (4) What categories of genes do phasiRNAs regulate? and (5) What is the broad spectrum of biological processes that may be associated with or regulated by phasiRNA biogenesis? To address these questions, we performed a systematic search of *PHAS* loci in 23 plant species covering major phylogenetic groups including alga, moss, gymnosperm, basal angiosperm, monocot, and dicot as shown in Fig. 1. Our study uncovered a wide range of novel *PHAS* loci from protein-coding genes. Intriguingly, many phasiRNAs appear to be derived from introns or intron-exon junctions. While some *PHAS* loci occur broadly in plants, many are found in a limited number of species. Production of phasiRNAs from protein-coding genes is associated with developmental processes, plant–virus interaction, and abiotic stresses, demonstrating the highly dynamic nature of phasiRNA biogenesis regulation. Our data provide the most comprehensive kingdom-wide landscape to date of phasiRNA distribution and potential regulatory function in plants, which will serve as an important foundation for developing mechanistic studies on the evolution, biogenesis, and function of these novel siRNAs underlying many aspects of plant biology.

## Results and discussion

### A broad search for *PHAS* loci in different plant species

We collected deep small RNA (sRNA) sequences of 23 plant species spanning from green algae to angiosperm groups from public resources (Fig. 1). These sRNAs were generated from organs or tissues at different developmental stages or under various growth conditions. The majority of the libraries contained 1–5 million high-quality cleaned sRNA sequences [27–33] (Additional file 1). To identify phasiRNA-generating loci, we aligned the sRNA sequences to the corresponding genome and cDNA reference sequences (Additional file 2). Based on the alignments, we identified *PHAS* loci from the 23 species using the method described in Xia et al. [23] (see Methods for details). The phasiRNA-generating loci mapped to the same protein-coding gene were further combined to represent a unique locus.

In total, we found 3,339 *PHAS* loci in all the 23 species, including 3,306 that generated 21-nucleotides (nt) and 33 that generated 24-nt phasiRNAs (Table 1). The 24-nt phasiRNA-generating loci were found exclusively in the grass family (*Oryza rufipogon*, *Setaria italica*, and *Sorghum bicolor*), and all of them were mapped to intergenic regions (Additional file 3). The number of 21-nt phasiRNA-generating loci in each plant group or species varied greatly (Additional file 4: Figure S1). Dicot species tended to have large numbers of *PHAS* loci, with many species having more than 100. In addition, approximately 75 % (1,122 out of 1,511 as listed in Table 1) of the *PHAS* loci mapped to annotated genic regions in all tested dicot species. The number of *PHAS* loci in monocot plants ranged from 13 to more than 900. In contrast to those in dicots, significantly fewer *PHAS* loci were mapped to genic regions in monocots. In a basal angiosperm species, *Amborella trichopoda*, there were 67 phasiRNA-generating loci, comparable to that in many dicot plants. In moss, *Physcomitrella patens*, there were four *PHAS* loci. In algae, *Chlamydomonas reinhardtii* had a comparable number of *PHAS* loci as in many higher plants whereas *Volvox carteri* generated phasiRNAs from only six loci. The complete list of 21-nt phasiRNA-generating loci is provided in Additional file 5. All the *PHAS* loci identified in the present study are stored in an interactive online database we developed [34]. The database provides a set of query interfaces and tools to analyze, visualize, and mine the phasiRNA data presented in this study.

**Table 1:** Summary of *PHAS* loci identified from various plant species

Species	Protein-coding gene				Intergenic	Total
	Exon	Intron	Exon-intron	Subtotal		
21-nt phasiRNA generating loci						
*Populus trichocarpa* (poplar)	125	3	9	137	12	149
*Citrus sinensis* (sweet orange)	233	1	25	259	97	356
*Arabidopsis lyrata*	27	1	4	32	19	51
*Arabidopsis thaliana*	33	0	0	33	21	54
*Carica papaya* (papaya)	104	3	15	122	33	155
*Vitis vinifera* (grape)	132	24	77	233	123	356
*Nicotiana tabacum* (tobacco)	15	1	4	20	6	26
*Solanum tuberosum* (potato)	146	1	9	156	45	201
*Solanum lycopersicum* (tomato)	87	9	16	112	11	123
*Mimulus guttatus* (monkey flower)	16	0	2	18	22	40
*Musa acuminata* (banana)	18	0	5	23	6	29
*Oryza sativa* ssp. *japonica* (rice)	25	4	4	33	23	56
*Oryza sativa* ssp. *indica* (rice)	6	2	2	10	6	16
*Oryza rufipogon* (wild rice)	5	3	1	9	4	13
*Panicum virgatum* (switchgrass)	10	0	0	10	14	24
*Setaria italica* (foxtail millet)	16	1	1	18	898	916
*Zea mays* (maize)	21	0	1	22	239	261
*Sorghum bicolor* (sorghum)	40	2	6	48	19	67
*Amborella trichopoda*	19	2	2	23	42	65
*Cycas rumphii*	12	0	0	12	1	13
*Ginkgo biloba*	25	0	0	25	0	25
*Physcomitrella patens*	0	0	0	0	4	4
*Volvox carteri*	5	0	2	7	20	27
*Chlamydomonas reinhardtii*	126	34	49	209	70	279
24-nt phasiRNA generating loci						
*Oryza rufipogon* (wild rice)	0	0	0	0	7	7
*Setaria italica* (foxtail millet)	0	0	0	0	23	23
*Sorghum bicolor* (sorghum)	0	0	0	0	3	3

### Conserved and unique *PHAS* loci from non-coding DNA sequences

The non-coding *TAS* loci (*TAS1*, *TAS2*, *TAS3*, and *TAS4*) were first identified in *Arabidopsis thaliana*. miR173 is responsible for triggering *TAS1* and *TAS2*, which seems to be unique in *A. thaliana* [11, 35]. In contrast, *TAS3* and its cognate trigger miR390 were found from moss to most of the land plants examined [12]. Our searches in 23 plant species confirmed that *TAS1* was unique to *A. thaliana*, *TAS2* could only be found in *A. thaliana* and *A. lyrata*, and *TAS3* was present in many land plant species.

The taxonomic distribution of *TAS4* and its cognate trigger miR828 was enigmatic. Previous studies postulated that the miR828–*TAS4* regulatory circuit might exist only in *Arabidopsis thaliana* and a few closely related species, but the possibility of a wider distribution was proposed based on the presence of miR828 and/or *TAS4* homologs in several other plant genomes [35]. Our searches identified bona fide *TAS4* loci based on the production of phasiRNAs in several dicot species, including poplar (*Populus trichocarpa*), sweet orange (*Citrus sinensis*), grape (*Vitis vinifera*), tomato (*Solanum lycopersicum*), tobacco (*Nicotiana tabacum*), potato (*S. tuberosum*), and monkey flower (*Mimulus guttatus*) (Additional file 6). The miR828–*TAS4* regulatory circuit was suggested to play a role in trichome development in *A. thaliana* [36] and apple [20], fiber growth in cotton [36], flavonoid biogenesis in *A. thaliana* [37], and perhaps fruit development in tomato [38]. In line with previous reports [35], we did not detect the presence of miR828 or *TAS4-*derived phasiRNAs in any of the tested monocot species. Interestingly, although the *MIR828* gene could be found in the genomes of all test species, its reads were only found in poplar and grape sRNA libraries (both have miR828 reads below 5 RPM) (Additional file 6), in contrast to the detection of highly abundant miR828 in cotton [36]. It is possible that the miR828 level was too low to be detected in sRNA libraries from many plant species investigated in this study.

Intriguingly, we found a miR828 homolog in the basal angiosperm *Amborella trichopoda* (Additional file 4: Figure S2A) based on the homology of mature miRNA sequences. This miRNA was predicted to trigger phasiRNA production from an intergenic region. It is noteworthy that phasiRNA production triggered by Atr-miR828 in *Amborella trichopoda* did not yield the conserved D4(−) sequence (Additional file 4: Figure S2B) that was found in *Arabidopsis thaliana* and many other species [35]. Our current data are insufficient to draw a conclusion about the phylogenetic relationship between the miR828–*PHAS* regulatory circuit in *Amborella trichopoda* and that in dicots, but raise the interesting possibility that the *Amborella trichopoda* circuit was a prototype that has stabilized and been refined in dicots but been lost in monocots during evolution.

Our searches uncovered many *PHAS* loci from noncoding sequences from various species (Additional files 3 and 5) and we did not analyze them in further detail. Our subsequent analyses were devoted to *PHAS* loci derived from protein-coding genes.

### An expansive and diverse repertoire of *PHAS* loci originated from protein-coding genes

Our analyses identified approximately 1,600 protein-coding genes, belonging to 217 categories (Additional file 7) based on their functional annotations, that serve as *PHAS* loci in at least one species, significantly expanding the list of 119 currently identified gene categories that can produce phasiRNAs (Additional file 8). These *PHAS* loci cover a wide range of genes, which include those encoding disease resistance and wound-response proteins, hormone response factors, transcription factors, RNA silencing components, proteins involved in signal transduction, transporters, protein translation machinery components, photosystem components, histone and DNA methylation proteins, the cytoskeleton and associated factors, intracellular trafficking machinery, kinases, and other enzymes involved in diverse metabolic pathways. (Additional file 7). Transcription factors and kinases were also found to function as *PHAS* loci in soybean [16].

When the total number of *PHAS* loci from leaves of land plants was compared, some interesting patterns emerged (Additional file 4: Figure S3 and Additional file 9). The eudicots and basal angiosperm *Amborella trichopoda* had relatively higher numbers of *PHAS* loci than monocots. Among eudicots, sweet orange had the largest number of *PHAS* categories from the annotated gene loci in leaves. Grapevine and poplar also had a large number of *PHAS* loci from annotated gene loci in leaves. Closely related species may have different protein-coding *PHAS* loci enriched. For example, PPR-containing protein genes were the dominant *PHAS* loci in *Arabidopsis thaliana* leaves, whereas TIR-NBS-LRR class disease resistance genes were the most enriched in *Arabidopsis lyrata* leaves.

Disease resistance genes, including NBS-LRR genes, constitute the most conserved group of *PHAS* loci, confirming and expanding previous reports [15, 16, 23]. Our extensive search found phasiRNAs produced from NBS-LRR genes in gymnosperms, the basal angiosperm *Amborella trichopoda* and monocots, although much less than in dicots (Additional files 5 and 7). Thus, disease resistance genes evolved as *PHAS* loci in early seed plants but were strongly selected for in dicot plants. Expression of R genes constitutes a high cost for plants [39]. Infection of several viruses and a bacterium which encode silencing suppressors led to down-regulated expression of miRNAs and siRNAs that trigger phasiRNAs from R genes [18], leading to the hypothesis that repressed expression of R genes by phasiRNAs under non-infection conditions functions to minimize the costs for plants [17, 18] and that viral or bacterial suppressor-induced expression of R genes enables or enhances plant defense responses during infection [18]. The wide taxonomic distribution of disease resistance genes as *PHAS* loci in our analysis lends further support to this hypothesis.

While some *PHAS* loci, such as disease resistance genes and Myb domain-containing genes, were widespread among the plants we examined, most loci were found in only a few species or just one single species. For example, a squamosal promoter-binding protein gene served as a *PHAS* locus only in grapevine though this gene family is a conserved target of miR156 in many plants. The stochastic occurrence of many *PHAS* loci in the plant kingdom suggests their young evolutionary origins and the existence of yet-to-be identified biogenesis factors. This, together with the diversity of protein-coding genes acting as *PHAS* loci, suggests phasiRNAs as the fastest-evolving riboregulators to regulate the expression of continuously expanding numbers and types of protein-coding genes. As such, this regulation may be an important contributor to the diversification of certain plant phenotypes.

### Broad taxonomic distribution of RNA silencing machinery components as *PHAS* loci

PhasiRNAs can be generated from RNA silencing machinery components, including *DICER-LIKE (DCL)*s and *SUPPRESSOR OF GENE SILENCING 3 (SGS3)* in *Medicago truncatula* [15], peach [23], and soybean [16], as well as *ARGONAUTE2 (AGO2)* in peach [23], and soybean [16]. We found *SGS3* to be a *PHAS* locus in leaf and cone samples of the two gymnosperm species examined (*Cycas rumphii* and *Gingko biloba*) and in leaf and fruit samples of grape. Interestingly, *Arabidopsis thaliana SGS3* did not produce any siRNAs. We also observed *DCL*-derived 21-nt phasiRNAs from papaya, sweet orange, tomato, and tobacco, *AGO*-derived 21-nt phasiRNAs from *A. thaliana*, *A. lyrata*, monkey flower, tomato and sorghum, and *RNA-DEPENDENT RNA POLYMERASE (RDR)*-derived 21-nt phasiRNAs from foxtail millet and *Amborella trichopoda*. These new observations, together with earlier reports [15, 16, 23], support the notion that phasiRNAs generated from RNA silencing machinery genes may function in a feedback mechanism to regulate the expression of these genes [15], and that this mechanism may be widespread in plants.

### Novel biogenesis of phasiRNAs from introns or exon-intron junctions

The noncoding *TAS2* locus in *Arabidopsis thaliana* produces siRNAs from an alternative transcript that retained an intron [40]. In the present study, we found a large number of protein-coding genes that generate phasiRNAs from annotated introns alone (Additional file 10) or intron–exon junctions (Additional file 11) in many plant species. For example, we found that phasiRNA production from the *SGS3* locus in grape progressed from an exon into an adjacent intron (Additional file 4: Figure S4A). In addition, a serine/threonine protein kinase gene produced phasiRNAs only from its second intron (Additional file 4: Figure S4B). In total, we identified 107 *PHAS* loci producing phasiRNAs only from annotated introns and 157 loci producing phasiRNAs from intron–exon junctions. To validate intron annotations, we used GBrowser in the Phytozome [41], Solgenomics [42], and *Amborella* genome [43] databases to examine expressed sequence tag and/or RNA-sequencing (RNA-Seq) data for these *PHAS* loci. The data validated annotations for approximately one third (34 out of 107) of the intron-only and one fourth (37 out of 157) of the intron–exon junction *PHAS* loci (Additional files 10 and 11). Intriguingly, the majority of *PHAS* loci that generated phasiRNAs from introns or intron–exon junctions were disease resistance genes (Additional file 4: Figure S5, and Additional files 10 and 11).

**Fig. 2:**
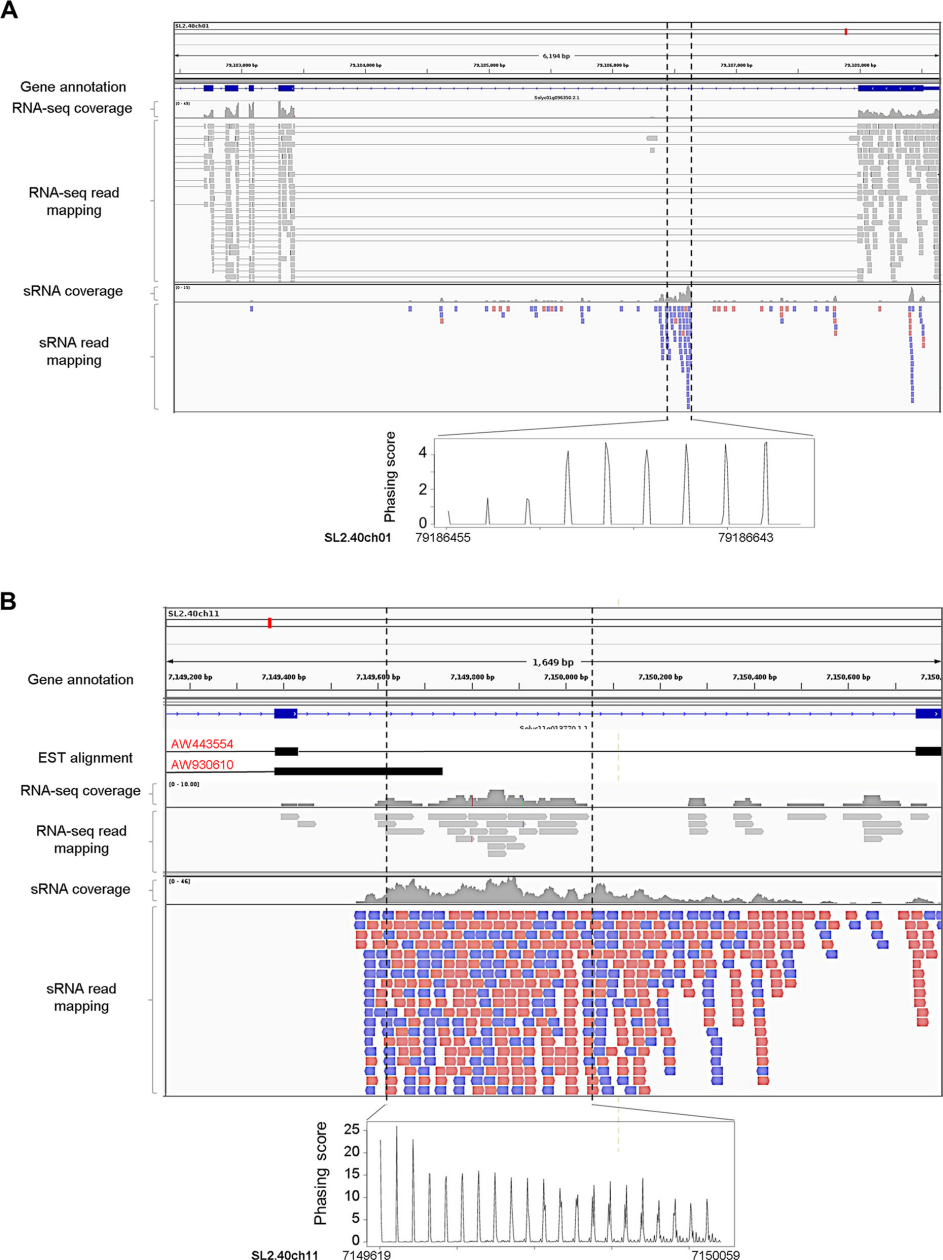
PhasiRNAs generated from introns in tomato. Examples of a *PHAS* locus (*Sly015*) that generates phasiRNAs from a bona fide intron (**a**) and a *PHAS* locus (*Sly110*) that generates phasiRNAs from an alternatively retained intron (**b**). RNA-seq and sRNA reads were derived from the same organ (fruit at the breaker stage or 42 days after pollination) and mapped to the tomato genome. For sRNA mapping, *red* depicts reads aligned in forward orientation and *blue* in reverse orientation. *EST* expressed sequence tag

An outstanding question is whether phasiRNAs from the intron-only *PHAS* loci are generated from free-standing introns or from alternatively spliced or aberrant transcripts. We addressed this question by analyzing the published tomato dataset that contains RNA-seq and sRNA data from the same samples [44]. We found three annotated intron regions that generated phasiRNAs but had no RNA-seq coverage, indicating that they are bona fide introns. An example is shown in Fig. 2a. We also found 11 annotated intron regions, which generated phasiRNAs with good RNA-seq coverage, indicating that they are retained in the alternatively spliced transcripts (an example is shown in Fig. 2b). These data provide experimental evidence that free-standing introns or introns retained in the alternatively spliced transcripts can indeed be the direct sources of phasiRNAs.

It remains unclear whether the phasiRNAs from free-standing introns are generated via the conventional phasiRNA biogenesis pathway where a miRNA trigger functions in the cytoplasm or via a novel pathway operating in the nucleus. Regardless of the specific pathway(s), our findings revealed an unexpected link among mRNA splicing, mRNA stability, and phasiRNA regulation. This might represent a new mechanism of removing aberrant (i.e., unspliced or incorrectly spliced) RNA transcripts that escape the nucleus to accumulate in the cytoplasm. Alternatively, phasiRNA biogenesis from introns and/or intron–exon junctions of certain loci may function to regulate pre-mRNA stability or other aspects of gene expression.

### Unique developmental regulation of *PHAS* expression in different plants

Comprehensive analysis of phasiRNAs in soybean identified a number of tissue-specific *PHAS* loci [16]. Most plant species we analyzed had sRNA libraries prepared from different organs and/or from various developmental stages, thereby allowing examination of the developmental significance of phasiRNA generation among different taxonomic groups. It is worth noting that sRNA libraries we used for comparative analysis for a given plant were all derived from the same genotype and generated by the same laboratory, and have been used to explore the widespread conservation and divergence of microRNAs in plants [33]. Our analyses showed that many protein-coding genes that acted as *PHAS* loci in one organ did not produce any siRNAs or produced only abundant non-phased siRNAs in another organ(s) of the same species (Additional file 12). For example, genes encoding NAC domain-containing transcription factors were an obvious cluster in producing phasiRNAs in fruits but much less in leaves and flowers of sweet orange (Additional file 4: Figure S6), implying their unique roles in regulating sweet orange fruit development.

**Fig. 3:**
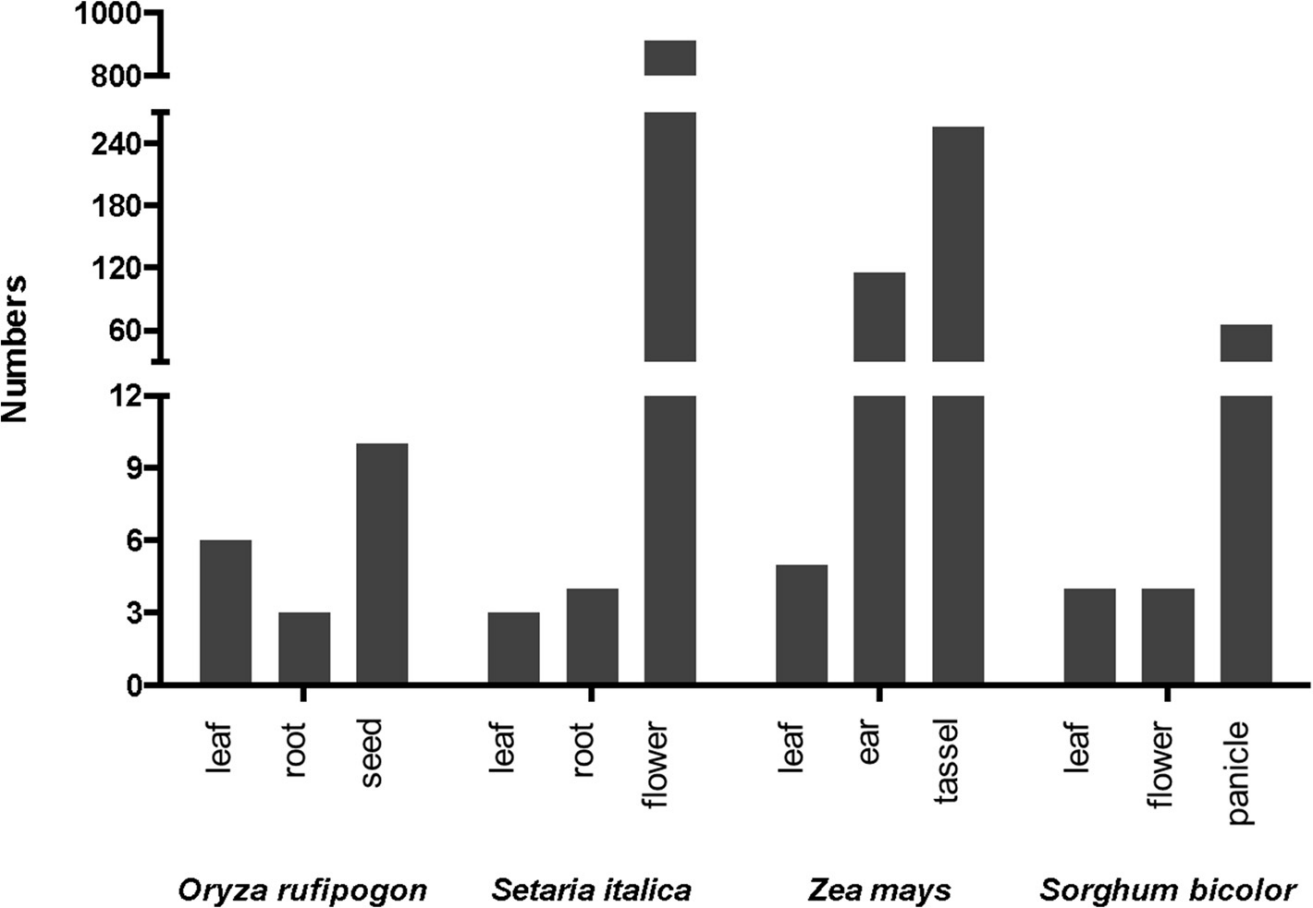
Number of *PHAS* loci identified in different organs of representative species in the grass family

All tested monocot plants possessed very few loci for generating 21-nt phasiRNAs in non-reproductive organs such as leaf, shoot, and root. For species in the grass family, there was a drastic induction of the *PHAS* loci in certain reproductive organs, such as the flower of foxtail millet, ear and tassel of maize (*Zea mays*), and panicle of sorghum. Furthermore, most of these *PHAS* loci were in the intergenic regions as shown in Fig. 3 and Additional file 12.

Notably, wild rice had five *PHAS* loci producing phasiRNAs only in the seeds, but no siRNAs in the shoots or roots. Among these five loci, a phosphoinositide phosphatase family gene and a nuclear factor Y gene produced 20-fold more phasiRNAs than those generated by a deeply conserved noncoding *PHAS* (*TAS3*). Thus, the regulatory roles of these two loci may help us understand the function of protein-coding transcript-derived phasiRNAs in cereal development.

These observations, together with the findings from Arikit et al. [16], indicate that *PHAS*-based gene regulations are unique in different organs in a wide range of plants. Interestingly, organ-specific *PHAS* loci did not exhibit any general conservation among different plant species. Whether this may contribute to the development of distinct phenotypes in different plants remains an outstanding question.

It should be noted that the datasets we have analyzed for land plants were mostly derived from whole plant organs. A recent study reported phasiRNAs in maize anther and pollen at different developmental stages, providing deeper insights into phasiRNA biogenesis at the tissue-specific level [45]. Thus, future studies to analyze tissue- or even cell-specific expression patterns of phasiRNAs in different plants should shed new light on the functions of phasiRNAs in plant development processes.

### Viral infection induces or suppresses a diverse array of *PHAS* loci

Some miRNAs act as master regulators to initiate the generation of phasiRNAs from mRNAs that encode NBS-LRR disease resistance proteins in legumes [15, 16] and solanaceous plants [17, 18]. Viral and bacterial infection represses the expression of miRNA triggers and consequently also phasiRNAs from some resistance gene loci [17, 18]. To gain further insight into the potential role of phasiRNAs in plant–microbe interactions, we analyzed the global expression patterns of phasiRNAs in several virus–plant systems. Specifically, we examined phasiRNA profiles in healthy and *Papaya ringspot virus* (PRSV)-infected leaves of papaya (*Carica papaya*) [33], healthy and *Turnip mosaic virus*-infected leaves of *Arabidopsis thaliana* [29], as well as healthy and *Rice stripe virus* (RSV)- and *Rice dwarf virus* (RDV)-infected shoots of rice (*O. sativa* L. ssp*. Japonica*) [31] (see Additional file 1 for the source of sRNA data).

**Fig. 4:**
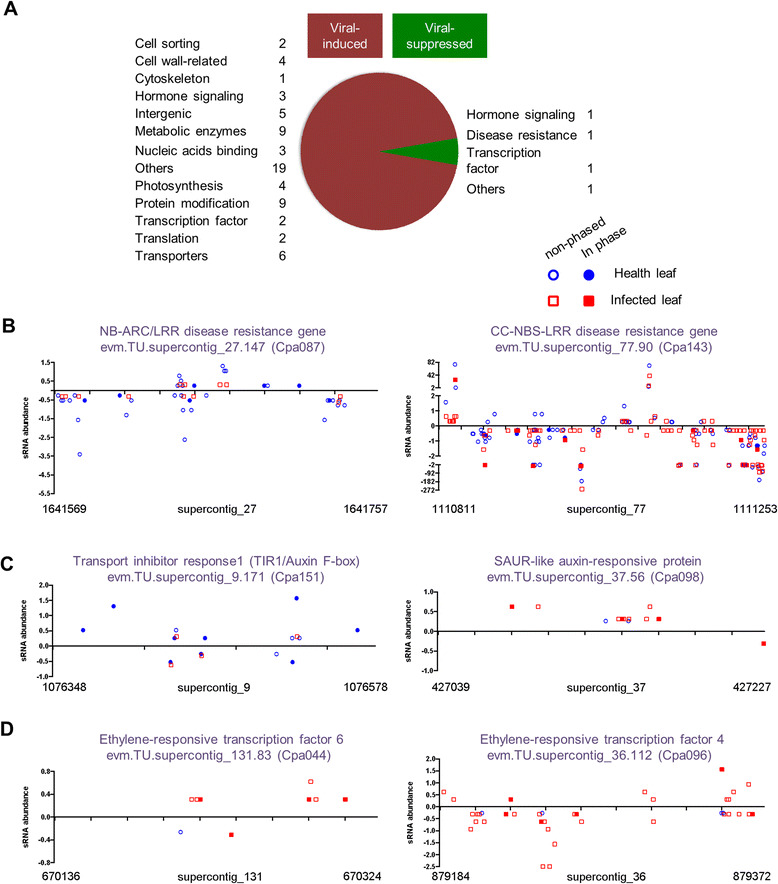
siRNA production from various *PHAS* loci upon viral infection. **a** Pie diagram showing the *PHAS* loci with more than five-fold changes in total 21-nt siRNA production in papaya leaves infected by *Papaya ringspot virus* as compared to that in healthy leaves. The number of *PHAS* locus or loci for each gene category showing induced or suppressed expression is indicated. **b**–**d** Specific examples of *PHAS* loci derived from disease-resistant genes (**b**), auxin-related genes (**c**), and ethylene-responsive transcription factor genes (**d**), which showed differential expression of siRNAs in healthy and infected leaves

In the papaya system, 40 and 93 *PHAS* loci were found in healthy and infected leaf libraries, respectively; among them, 13 were shared while many exhibited different expression patterns (Additional file 13). Six disease resistance *PHAS* loci showed reduced production of 21-nt siRNAs upon PRSV infection (13, 24, 33, 36, 62, and 87 %, respectively, of that in the healthy leaves). Five of them became non-*PHAS* loci in the infected leaves (Fig. 4b and Additional file 13). Furthermore, three out of four *PHAS* loci derived from auxin-signaling genes in the healthy leaves also became non-*PHAS* loci in the infected leaves: *ARF3* had the highest degree of total siRNA reduction in infected leaves and two auxin-signaling F-box genes (*AFB2* and *TIR1/AFB*) produced four times less siRNAs in infected leaves (Fig. 4c and Additional file 13).

Similarly in the rice and Arabidopsis systems, a large number of *PHAS* loci showed substantial changes in phasiRNA generation upon infection by different viruses (Additional file 13). It is noteworthy that an auxin response factor (ARF) gene in rice showed more than 8- and 3.5-fold changes in phasiRNA generation upon RSV and RDV infections, respectively.

While the repressed expression of some *PHAS* loci in the infected leaves was consistent with earlier reports in Solanaceae and Fabaceae [15–18], our analyses identified many novel *PHAS* loci induced by PRSV infection. These could be clustered into several groups based on their gene functions: cytoskeleton-related genes, chloroplast-related genes, cell wall-related genes, transporter genes, and hormone-signaling genes (Additional file 13). As an example, a SAUR-like ARF increased siRNA production by more than eight-fold and became a *PHAS* locus in infected leaves (Fig. 4c). Furthermore, two ethylene-responsive transcription factor genes were highly induced as *PHAS* loci in response to viral infection (Fig. 4d). Induced expression of these *PHAS* loci may function as part of the coordinated plant defense responses, by reducing metabolic activities to dampen viral replication. Many studies have reported up- or down-regulated expression of host genes during viral infection, with many types of genes appearing to be commonly affected in different host–virus systems [46–50]. The underlying molecular mechanisms are largely unknown. We suggest that viral infection-induced or suppressed expression of selective phasiRNAs may act as a novel mechanism to regulate the expression of selective host genes.

### Nutrient stresses modulate phasiRNA biogenesis in algae

Previous reports showed the presence of phasiRNAs in unicellular alga *C. reinhardtii*, mainly mapped to intergenic regions [51, 52]. In the present study, we identified more than 300 *PHAS* loci in this alga, of which approximately 240 were mapped to protein-coding genes. Among the protein-coding *PHAS* loci, nine were uniquely induced by sulfate starvation, five were uniquely induced by phosphate starvation, and one was induced by both treatments. On the other hand, six protein-coding *PHAS* loci were suppressed by both treatments, and four were uniquely suppressed by sulfate treatment (Additional file 4: Figure S7 and Additional file 14). It is noteworthy that there was no unique *PHAS* locus suppressed by phosphate starvation.

The biological implications of these changes in *PHAS* activities remain to be understood, but the suppressed expression of transposon-polyprotein loci is of notable interest. In *C. reinhardtii*, a *DCL1* homolog has been shown to repress retrotransposon activity [53]. However, the underlying molecular mechanism is unknown. In the case of REM1 Ty3-*gypsy* retrotransposon silencing, retrotransposon-derived siRNAs were postulated to be derived from double-stranded RNAs formed via a transposon polyprotein gene and its adjacent inverse transcripts [54]. However, we did not find evidence for the existence of such adjacent inverse transcripts that would contribute to the production of any siRNAs. Thus, phasiRNA-mediated retrotransposon silencing by phasiRNAs derived from the coding transcripts within the retrotransposon itself may be a novel mechanism underlying the retrotransposon silencing phenomenon in *C. reinhardtii*.

The multicellular alga *V. carteri* had much fewer *PHAS* loci. We found only one protein-coding *PHAS* locus in this alga growing under normal conditions. In contrast, five and one new protein-coding *PHAS* loci emerged under sulfate and phosphate starvation conditions, respectively.

Our analysis suggested that algae can respond to different abiotic stresses by differential gene expression modulated by phasiRNA biogenesis. In soybean, drought stress induced the expression of phasiRNAs from a protein-coding gene, *ALLENE OXIDE SYNTHASE* [16]. Future studies will determine whether dynamic changes in phasiRNA biogenesis may occur broadly in land plants in response to nutrient and other abiotic stresses.

### sRNA triggers for phasiRNA biogenesis

We predicted sRNA triggers for *PHAS* loci mainly based on sequence complementarity (see Methods for details). We noticed that a large number of predicted triggers have a putative guided cleavage site falling out of the N × 21 bp phase windows (where N is an integer) to the positions of phasiRNAs. We discarded these out-of-phase triggers if they were not confirmed previously by other studies or not supported by the sPARTA [55] analysis described below. In total we could predict triggers for approximately 10 % of the *PHAS* loci we identified in this study (Additional files 15 and 16). Among these, we could find 170 miRNA triggers, among which 135 were known miRNAs (with 32 distinct sequences) and 35 putative miRNAs (with 21 distinct sequences) (Additional file 16). There are six species with available sequencing data from degradome libraries from similar sample sources/organs (Additional file 1). We used these data to test our trigger prediction with sPARTA [55]. The results are summarized in Additional file 16. For 41 triggers (4 in *Arabidopsis thaliana*, 4 in rice, 16 in tomato, 10 in grape, and 7 in *Amborella trichopoda*), the predicted cleavage sites were validated by degradome libraries. We could not validate any in *P. patens*, which may be attributed to the different sources for degradome and sRNA libraries. Further experimental studies are necessary to validate the remaining triggers.

sRNA triggers may work via the two-hit model (2_21_), in which two sites of the transcript are recognized by the same sRNA [11] or two different sRNAs [13, 15, 56], or via the one-hit model (1_22_), in which a single 22-nt sRNA directs the cleavage of the target [6, 15, 17, 18, 26]. The numbers for the predicted 21-nt and 22-nt sRNA triggers at 5′- and 3′-ends are summarized in Additional file 15. There were many cases in which we only found one 21-nt trigger at either 5′- or 3′-end, which may have been due to the limit of the algorithm in identifying imperfect alignments of sRNAs and targets.

Disease resistance genes are targeted by the miR482/miR2118 superfamily in generating phasiRNAs [18]. Other miRNA triggers, such as miR6024, miR6025, miR6027, and miR472, have also been reported [17, 19]. We found these miRNAs as triggers in various species. In addition, we predicted that miR3623 in grape was also involved in regulating phasiRNA production from disease resistance genes. Whether this suggests parallel evolution of species-specific regulators or suggests miR3623 as a relative or member of the miR482/miR2118 superfamily awaits further analysis. For NAC domain transcription factors, the predicted trigger was miR7122 in sweet orange but miR6445 in poplar. The evolutionary relationship between the MIR7122 gene and MIR6445 gene also remains unknown.

These observations show that an orthologous protein-coding gene in different plant species may be targeted by different miRNAs to produce phasiRNAs. However, because various miRNA triggers for phasiRNA biogenesis may have their own distinct expression patterns, an orthologous protein-coding gene may serve as a *PHAS* locus in distinct spatial-temporal manners in various species. For example, NAC domain transcription factor genes were enriched *PHAS* loci in the fruit sample in sweet orange and in the xylem sample from wood trunk in poplar. These observations reveal the highly complex evolutionary and functional regulatory networks of miRNAs and phasiRNAs.

### Predicted target genes of phasiRNAs

We also predicted the downstream targets for phasiRNAs present with more than 10 RPM in a given library (Additional file 17). It was evident that nearly all phasiRNAs had near-perfect matches to the genes in the same family as their originating loci. Interestingly, a recent report by Boccara et al. [19] showed that phasiRNAs derived from disease resistance genes negatively regulate a broad spectrum of disease resistance genes in *Arabidopsis thaliana*.

Our analyses identified some phasiRNAs that may target genes outside the gene families of their originating loci. In most species, such potentially *trans*-regulatory phasiRNAs accounted for only a small percentage of the total phasiRNAs, generally less than 10 % with exceptions when a stringent cut-off score (1.5) was used for target prediction. Thus, in contrast to the dominant *trans*-regulatory mode of miRNAs [6], our analysis suggests that phasiRNAs predominantly regulate protein-coding genes from which they are derived and genes in the same families of phasiRNA-generating genes. As discussed earlier, many *PHAS* loci may be of recent evolutionary origin given their stochastic occurrence in plants, superficially similar to the origin of young miRNAs. However, unlike the majority of young miRNAs that do not have predicted target genes and are therefore likely function-neutral [6], phasiRNAs are all likely functional because they may target at least the genes from which they are originated.

### Evolution of the phasiRNA biogenesis machinery

The stochastic presence of many *PHAS* loci in the species we analyzed raised questions concerning the conservation of their biogenesis machinery. Studies on the model plant *Arabidopsis thaliana* demonstrated that the generation of 21-nt phasiRNAs involves the coordinated actions of protein factors DCL4, AGO7, RDR6, and SGS3, in addition to the core players for miRNA biogenesis and function such as DCL1 and AGO1 [7]. Assuming this is an essential set of proteins for phasiRNA biogenesis in any plant, we determined their presence in different plants by searching the available genome sequences of 41 species, covering algae, mosses, monocots, and dicots. Gymnosperm plants were not included for lack of full genome sequences.

**Fig. 5:**
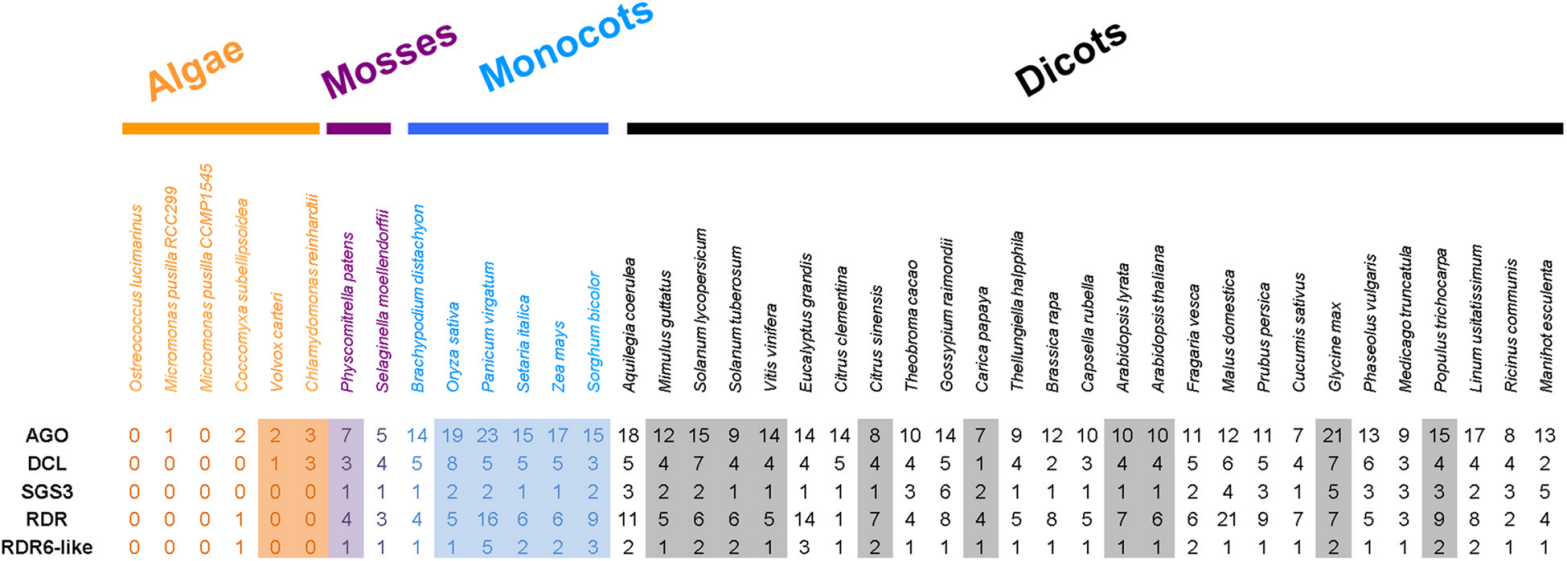
Summary of key protein factors involved in phasiRNA biogenesis in the plant kingdom. The *color shades* depict the species that were investigated for phasiRNA production in this study. The *numbers* listed indicate the copy numbers of the corresponding phasiRNA biogenesis genes

Our extensive search revealed that all proteins currently known to be specifically required for phasiRNA biogenesis were present in all of the tested land plants, from moss to higher plants. None of the algae species, single cellular or multicellular, had the complete set of SGS3, RDRs, DCLs, and AGOs. Among algae, only *C. reinhardtii* and *V. carteri* had dicer-like protein factors as shown in Fig. 5. Our search results match previous reports in several plant species including algae species [57], rice [58], maize [59], and tomato [60].

Thus, our results suggest that the currently known phasiRNA biogenesis pathway(s) has evolved only in land plants. These observations raise questions for future investigations: (1) what factors contribute to the stochastic evolution and activity of *PHAS* loci in different plant species, different developmental stages, and under different biotic and abiotic stress conditions; and (2) what machinery is responsible for the biogenesis of phasiRNAs in algae?

## Conclusions

Our extensive search across 23 different plant species uncovered a large and surprisingly diverse repertoire of protein-coding genes as novel *PHAS* loci. Biogenesis of phasiRNAs from many loci is uniquely associated with speciation, development, viral infection, or nutrient stresses. Numerous *PHAS* loci produced phasiRNAs from intron or exon–intron junction regions, revealing an unexpected link between phasiRNA biogenesis and RNA splicing. Our analysis suggests that phasiRNAs predominantly regulate protein-coding genes from which they are derived and genes in the same families of phasiRNA-generating genes, in contrast to the dominant *trans*-regulatory mode of miRNAs. Interestingly, while dicot *PHAS* loci appear to play an overwhelming role in disease resistance, monocot *PHAS* loci are mostly associated with reproductive growth.

The *PHAS* loci derived from protein-coding genes may be considered an evolutionary novelty in that these *PHAS* loci possess an added regulatory function specific to some plant species rather than a replacement of the original function of protein-coding genes for these loci. The stochastic occurrence of *PHAS* loci in the plant kingdom suggests their young evolutionary origins. The evolutionary flux that leads to high variations for a given locus among species or taxonomic groups suggests that phasiRNA networks may contribute to plant phenotype diversity by regulating distinct developmental processes or responses to biotic or abiotic stimuli.

In summary, our new findings, together with an interactive database developed, significantly expand previous findings and will help build a critical foundation to advance further studies on how phasiRNAs may have emerged to participate in the complex regulatory networks to shape the evolution of plant phenotype diversity.

## Methods

### sRNA sequence processing and alignment

sRNA deep sequencing data were downloaded from the Comparative Sequencing of Plant Small RNAs website [61] and the GenBank Gene Expression Omnibus [62] (Additional file 1). All sRNA deep sequencing data were treated via standard procedures for format conversion, adaptor trimming, and read collapsing. The treated sRNA sequences were further cleaned by removing sequences that matched rRNA sequences using Bowtie [63]. The cleaned sRNA sequences were mapped to the corresponding reference genome or transcriptome sequences (Additional file 2) using Bowtie [63], allowing no mismatches and no more than six hits to the reference sequences.

### Identification of candidate *PHAS* loci

We largely followed the methods developed by Xia et al. [23] for *PHAS* locus identification. Specifically, the mapped sRNA reads were denoted according to their positions in the corresponding reference genome and/or cDNA sequences. For matching sRNAs to the antisense strand of the reference sequences, a two-nucleotide positive offset was included to mimic the 3′ end overhang. A search was conducted by scanning transcriptome or genome references using a nine-phase register (21 bp as a phase register) sliding window (189 bp), each followed by a break of three-phase register (63 bp). A positive window was considered to contain no less than 10 unique reads, with more than half of the unique reads being 21 nt in length and with no less than three 21-nt unique reads falling into the phase registers. Windows were combined if they shared the same phase registers (i.e., the adjacent windows had a space of N × 21 bp, where N is an integer) and fell into the same genes.

Calculation of *P*-values for positive windows was based on the formula:

$$ \Pr \left(\mathrm{X}=\mathrm{k}\right)=\frac{\left(\begin{array}{c}\hfill 20m\hfill \\ {}\hfill n-k\hfill \end{array}\right)\left(\begin{array}{c}\hfill m\hfill \\ {}\hfill k\hfill \end{array}\right)}{\left(\begin{array}{c}\hfill 21m\hfill \\ {}\hfill n\hfill \end{array}\right)} $$

*P*-value: *p*(*k*) = ∑_*X* − *k*_^*m*^ Pr(*X*)

where n was the number of total unique 21-nt sRNAs matched within a window, k was the maximum number of unique 21-nt reads falling into one of the possible phase registers, and m was the number of 21-nt phases within a window. The 21-nt *PHAS* loci with a *P*-value of less than 0.001 were identified as the positive *PHAS* loci.

Phasing score was calculated following the methods described in de Paoli et al. [64] and Xia et al. [23] using the following formula:

$$ \mathrm{phasing}\kern0.5em \mathrm{score}\kern0.5em = \ln \left[{\left(1+10\times \frac{{\displaystyle {\sum}_{i-\mathrm{l}}^9 Pi}}{1+\sum U}\right)}^{k-2}\right] $$

where n was the number of phase registers occupied by at least one unique 21-nt sRNA within a nine-phase register window, *P* was the total number of reads for all 21-nt sRNA reads falling into a given phase in a given window, and U was the total number of unique reads for all 21-nt sRNAs falling out of a given phase.

The same formulas were applied to the 24-nt *PHAS* windows in all tested libraries, with a change of the phase register length from 21 nt to 24 nt. We used a cut-off phasing-score of 15 to identify positive 24-nt *PHAS* loci according to Johnson et al. [65].

### Prediction of *PHAS* triggers

PhasiRNAs are mainly triggered by 21-nt and/or 22-nt miRNA-mediated transcript cleavage in land plants. Occasionally, siRNAs could also serve as triggers. sPARTA [55] was used to search for the 21-nt/22-nt miRNA/siRNA triggers with relatively high abundance (>10 RPM in a given library) that potentially target 200 bp upstream and 200 bp downstream of a *PHAS* window with an alignment score cutoff of 4. The positive candidates were subject to Vienna RNA package [66] analysis to predict the miRNAs. The predicted miRNAs were then aligned with miRBase [67] registers (release 19) to unveil conserved miRNAs. sPARTA [55] was also employed to validate the miRNA-guided cleavage of transcripts from the cognate *PHAS* loci using the available degradome library data (Additional file 1). We removed the out-of-phase triggers that were not previously confirmed or were not supported by our sPARTA analysis.

### Prediction of phasiRNA targets

Target prediction for phasiRNAs was performed with sPARTA [55]. Twenty-one–nucleotide phasiRNAs with an abundance of no less than 10 RPM were used to predict their downstream targets. The alignment score cutoff was set to 3.

### Identification of protein factors responsible for phasiRNA biogenesis in plants

We followed the methods described in previous studies [58–60] with minor modifications to mine protein factors responsible for phasiRNA biogenesis in the genomes of 41 plant species. Briefly, the published amino acid sequences of each gene family from *Arabidopsis thaliana* were used to search against a plant genome database [41] using BLAST with an E-value cutoff of 1e-10. The candidates were examined for their functional domains, with those missing the essential domains removed. Specifically, for DCL homologs, we searched for candidates having high amino acid sequence homology (>50 % similarity) to the known DCLs and containing at least four of the six characteristic domains: DEAD, PAZ, Helicase-C, Duf283, RNase III, and double-stranded RNA-binding (DRB). We excluded those that do not have RNase III, PAZ, or Helicase-C domains. For AGO candidates, we looked for those having high sequence similarity (>50 %) to known AGOs and all three functional domains: DUF1785, PIWI, and PAZ. For RDRs in *A. thaliana*, RDR6 or RDR2 share little sequence similarity to RDR1, RDR3, RDR4, and RDR5, so we used RDR1, RDR2, and RDR6 individually as baits to search for candidates that shared high amino acid sequence homology and contained an RDR functional domain. For SGS3, we searched for those candidates having high amino acid sequence homology (>50 % similarity) to *A. thaliana* SGS3 and containing only XS zinc finger and XS domains.

## Additional files

Additional file 1: **Summary of small RNA and degradome RNA libraries.**

Additional file 2: **Reference genome and transcriptome databases used in this study.**

Additional file 3: **List of 24-nt phasiRNA generating loci.**

Additional file 4: Figure S1. Number of *PHAS* loci identified from all sRNA libraries in each species. **Figure S2.** Identification of Atr-miR828 and TAS4-like sequences in *Amborella trichopoda*. **Figure S3.** Number of *PHAS* loci identified in leaf samples of each species. **Figure S4.** phasiRNAs mapped to intron–exon junctions or to an intron alone. **Figure S5.** Numbers of *PHAS* loci mapped to introns or intron–exon junctions in different plant species. **Figure S6.** Developmentally regulated phasiRNA production. **Figure S7.***PHAS* loci in response to nutrient starvation in *Chlamydomonas reinhardtii*.

Additional file 5: **List of 21-nt phasiRNA generating loci.**

Additional file 6: **List of phasiRNA-generating TAS4 loci.**

Additional file 7: **Number of PHAS loci mapped to protein-coding genes.**

Additional file 8: **List of previously reported gene families that generate phasiRNAs.**

Additional file 9: **Summary of** ***PHAS*** **loci identified in each small RNA library.**

Additional file 10: ***PHAS*** **loci containing only intron-derived sRNAs.**

Additional file 11: ***PHAS*** **loci containing both intron- and exon-derived sRNAs (i.e., at intron–exon junctions).**

Additional file 12: **Developmentally controlled** ***PHAS*** **loci.**

Additional file 13: **Differential phasiRNA production in papaya,** ***Arabidopsis*** **, and rice between healthy and virus-infected leaf samples.**

Additional file 14: ***PHAS*** **loci that were responsive to nutrient starvation.**

Additional file 15: **Summary of** ***PHAS*** **loci with identified triggers.**

Additional file 16: **Analysis for** ***PHAS*** **loci triggers.**

Additional file 17: **Complete list of predicted target genes of phasiRNAs with RPM value no less than 10.**
