# Supplementary material for: A dynamic evolutionary and functional landscape of plant phased small interfering RNAs
Source: BMC Biol. 2015 May 16;13:32. doi: 10.1186/s12915-015-0142-4 (PMC4457045; doi:10.1186/s12915-015-0142-4)
Supplement: Supplementary file 4 — Number of PHAS loci identified from all sRNA libraries in each species. Figure S2. Identification of Atr-miR828 and TAS4-like sequences in Amborella trichopoda. Figure S3. Number of PHAS loci identified in leaf samples of each species. Figure S4. phasiRNAs mapped to intron–exon junctions or to an intron alone. Figure S5. Numbers of PHAS loci mapped to introns or intron–exon junctions in different plant species. Figure S6. Developmentally regulated phasiRNA production. Figure S7.PHAS loci in response to nutrient starvation in Chlamydomonas reinhardtii. [file 12915_2015_142_MOESM4_ESM.pdf]

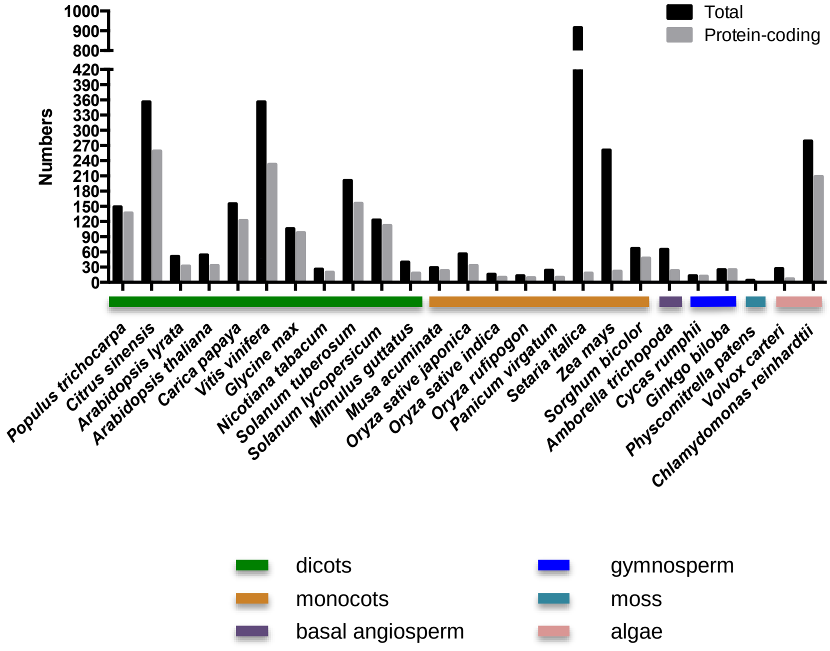

Figure S1. Number of *PHAS* loci identified from all sRNA libraries in each species.

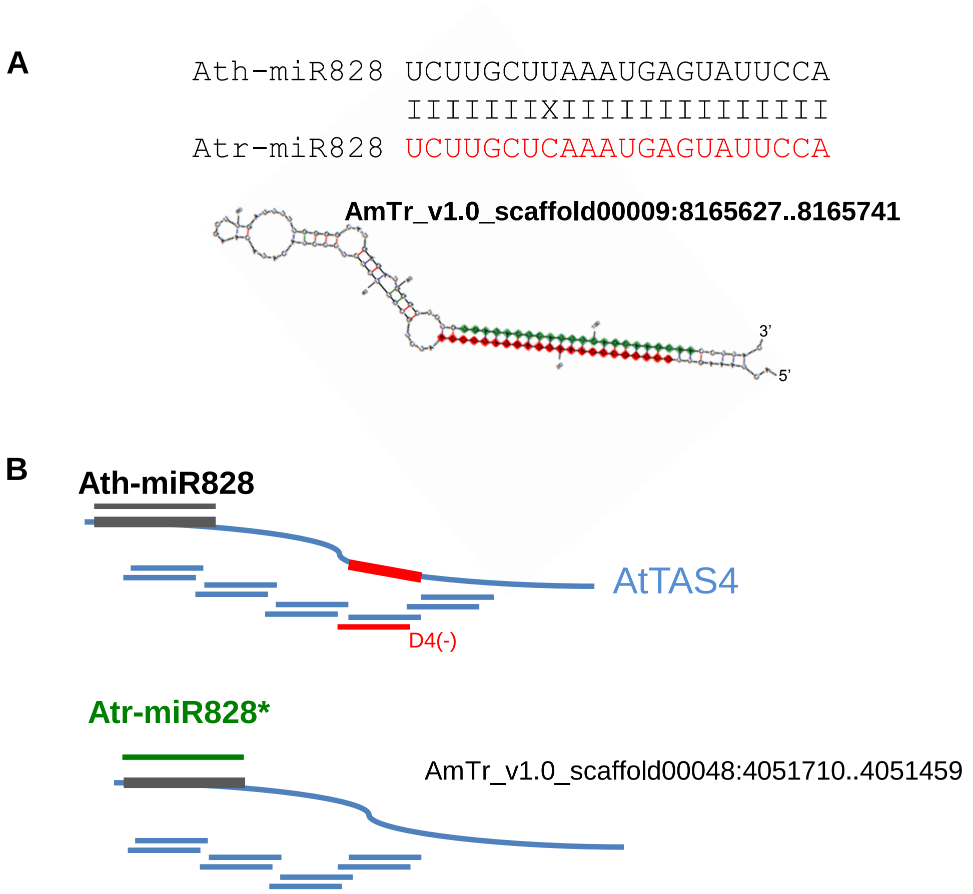

Figure S2. Identification of Atr-miR828 and TAS4-like sequences in *Amborella trichopoda*. (**A**) Sequence alignment of Atr-miR828 and Ath-miR828 and the folding of the Atr-miR828 precursor. Atr-miR828 sequences are highlighted in red and the corresponding Atr-miR828* sequences are highlighted in green in the folded structure. (**B**) Absence of a conserved D4(-) sequence in the TAS4-like sequence from *A. trichopoda*.

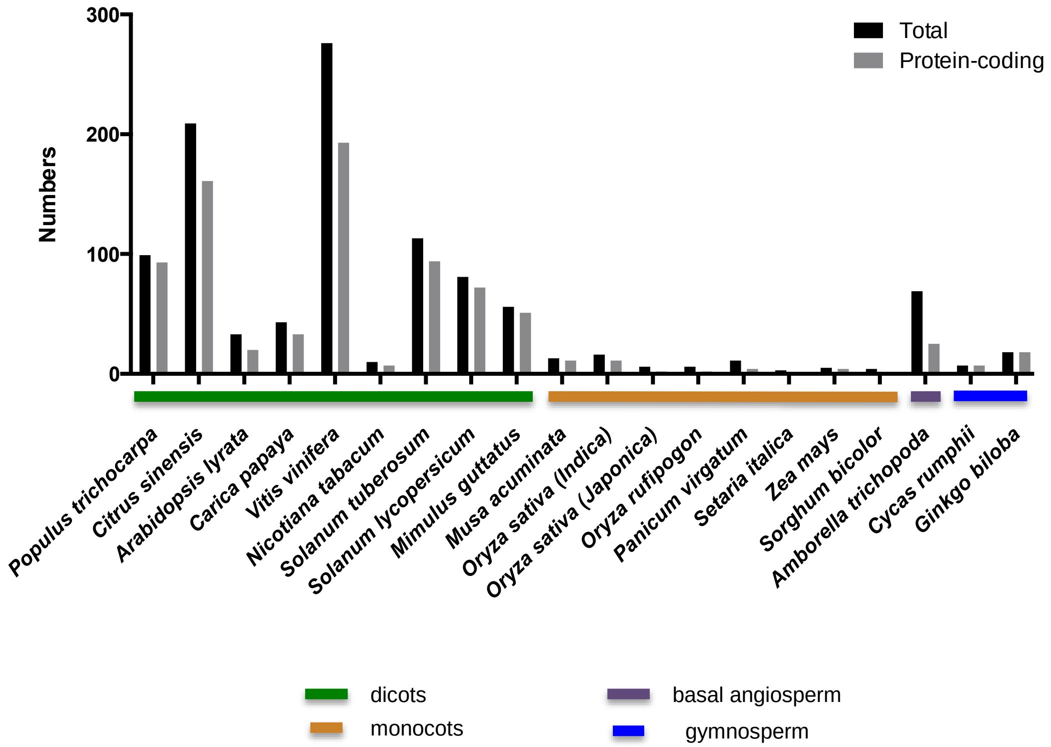

Figure S3. Number of *PHAS* loci identified in leaf samples of each species.

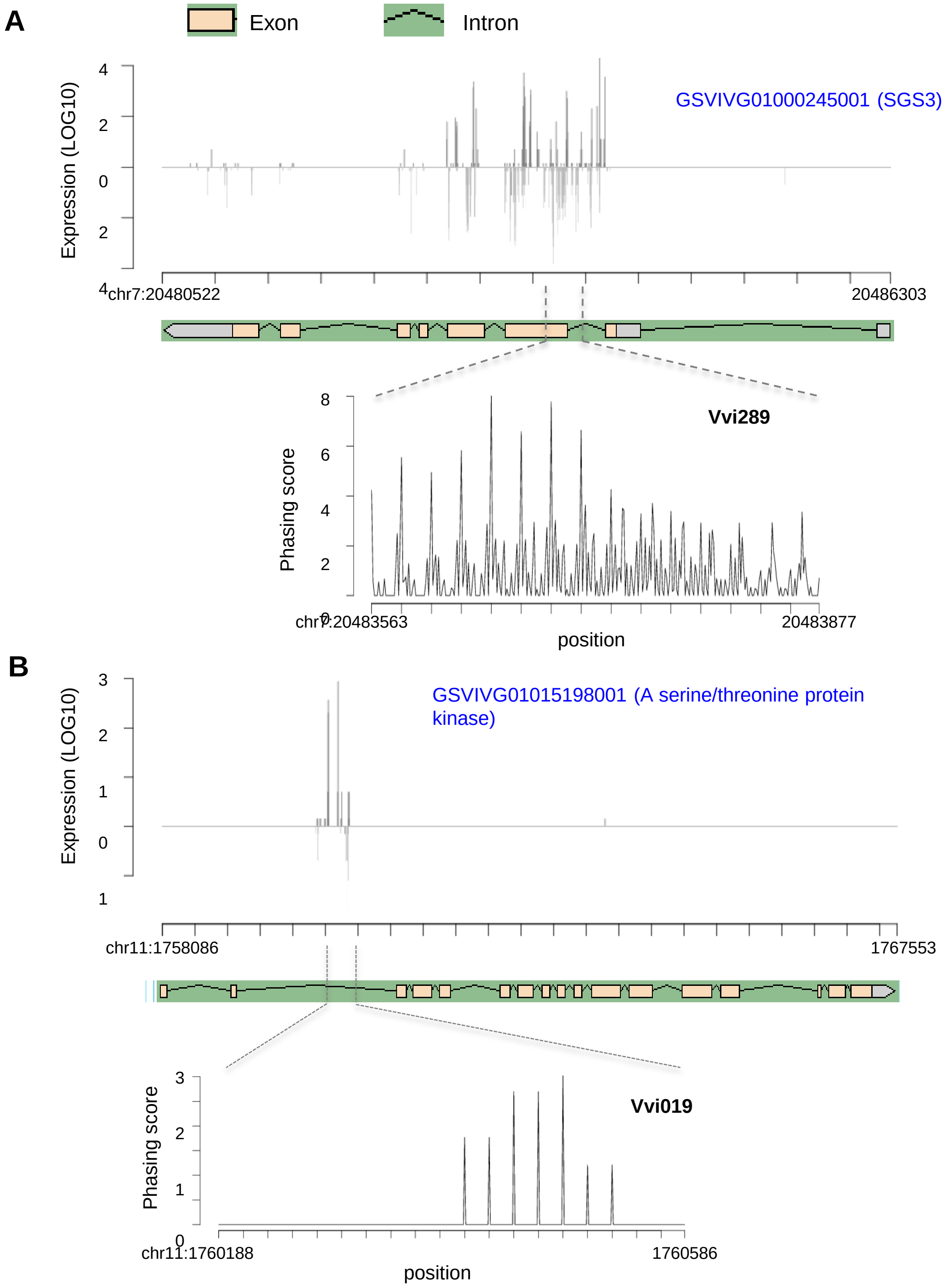

Figure S4. phasiRNAs mapped to intron-exon junctions or to an intron alone. (**a**) Example of a *PHAS* locus (a grape *SGS3* gene) that generates phasiRNAs across intron-exon junctions. (**b**) Example of a PHAS locus (a grape serine/threonine protein kinase gene) that generates phasiRNAs only from its second intron. Colored bars illustrate the gene model with introns and exons. Diagrams above the gene model show siRNA distribution and abundance (sense strand reads mapped to above the x-axis and the antisense strand reads are below the x axis). Diagrams below the gene model show phasing scores of the siRNAs.

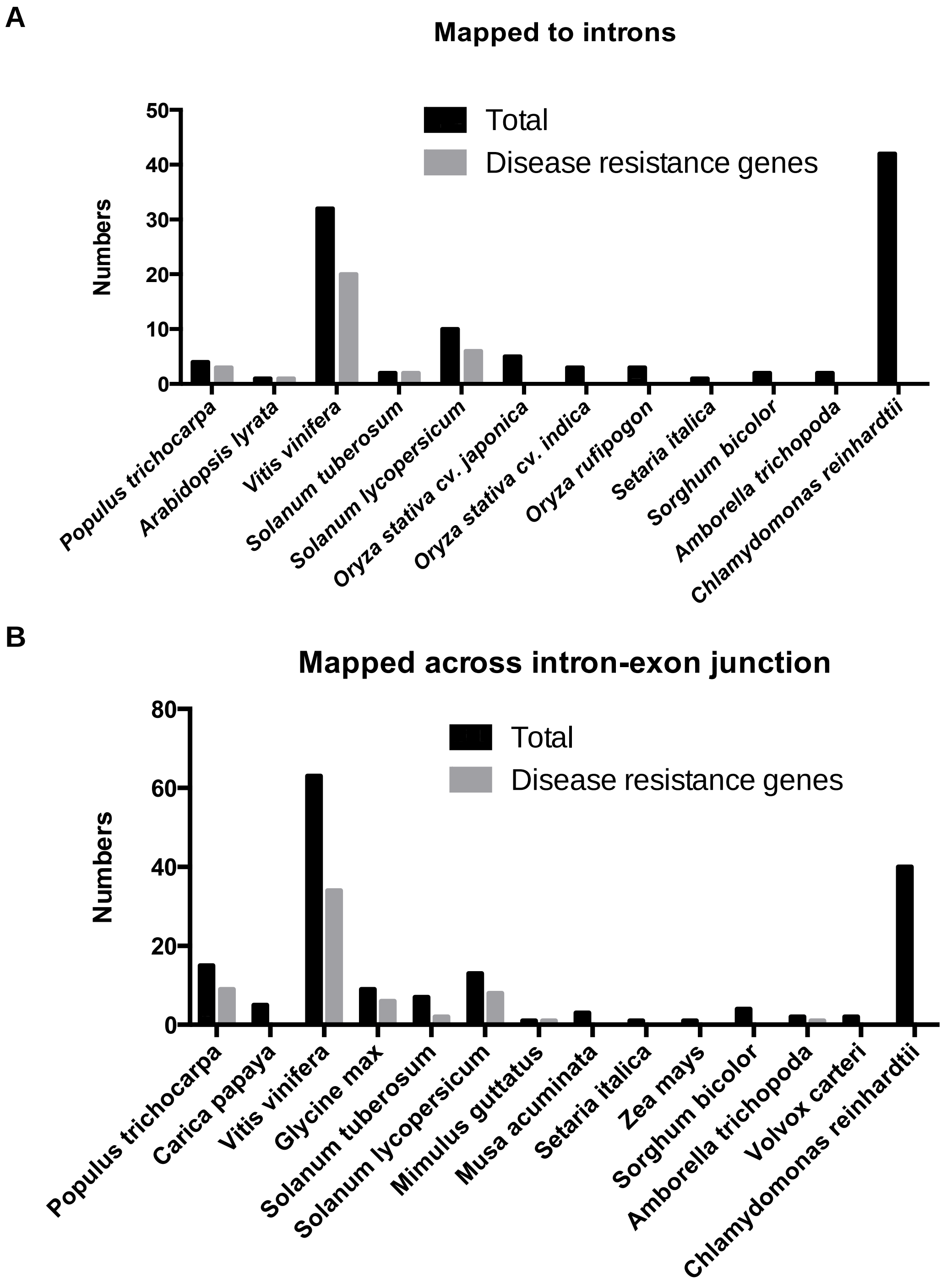

Figure S5. Numbers of *PHAS* loci mapped to introns (**a**) or intron-exon junctions (**b**) in different plant species.

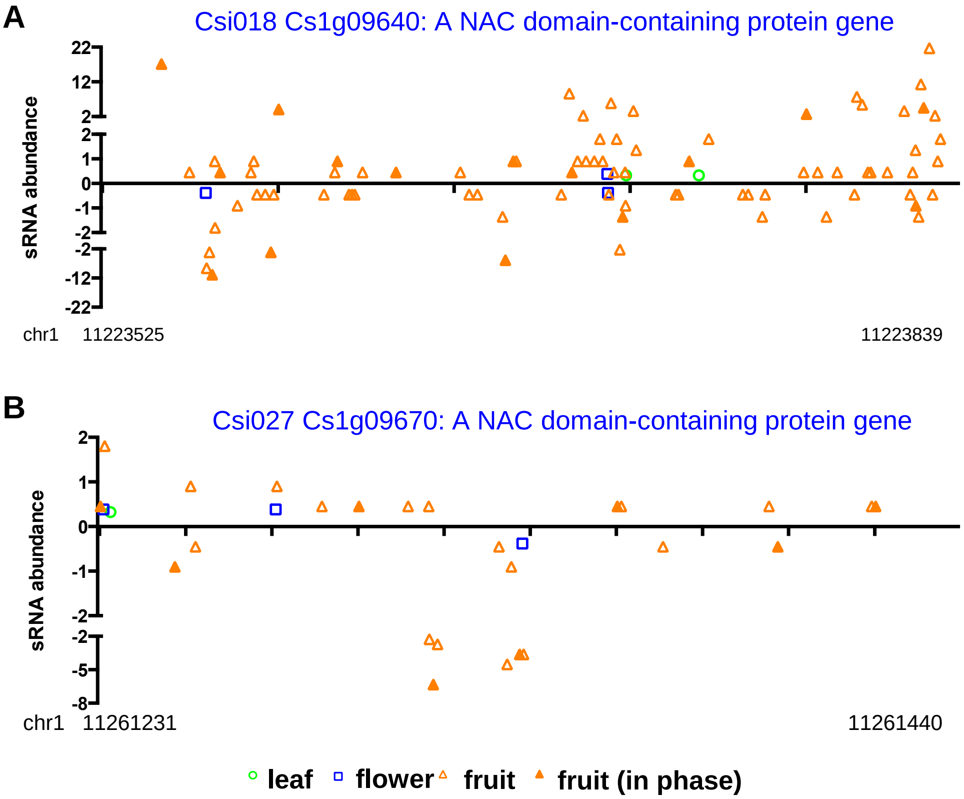

Figure S6. Developmentally regulated phasiRNA production. siRNA distribution and abundance (sense strand reads have positive values; antisense strand reads have negative values) at two sweet orange *PHAS* loci, *Csi018* (**A**) and *Csi027* (**B**), both derived from genes (*Cs1g09640* and *Cs1g09670*, *respectively*) encoding NAC domain-containing transcription factors.

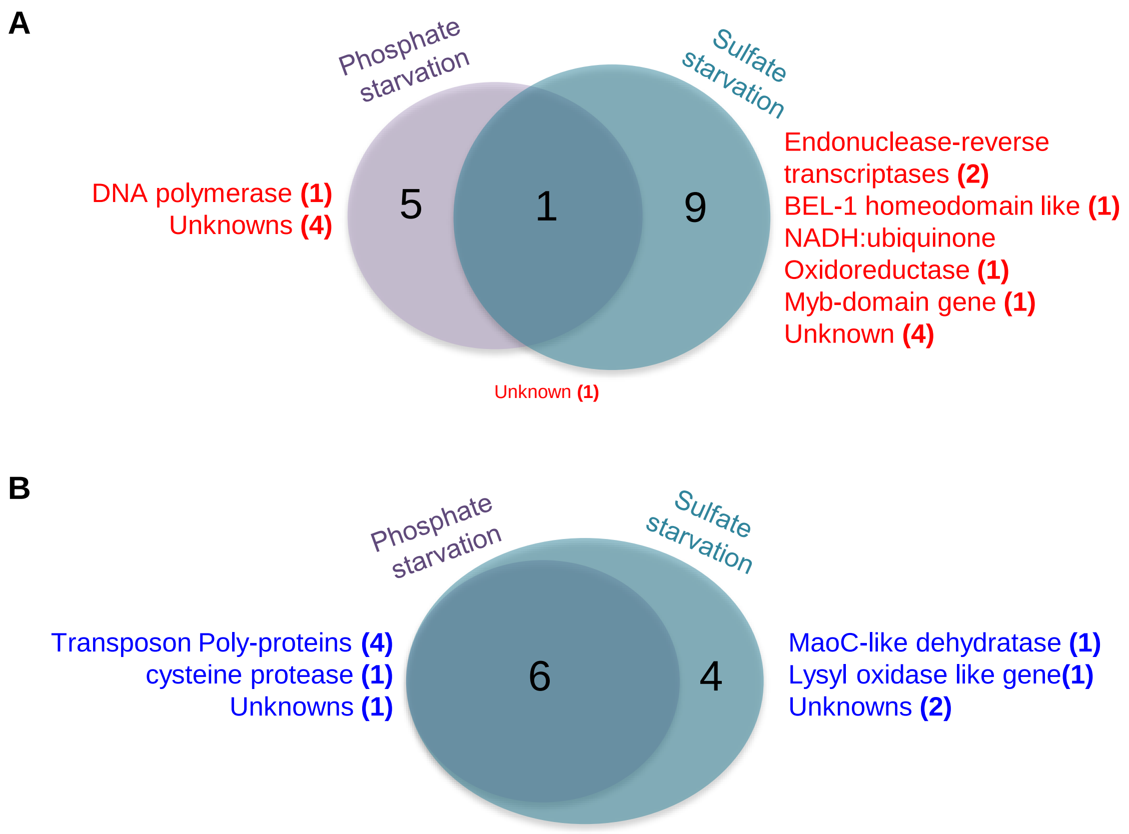

Figure S7. *PHAS* loci in response to nutrient starvation in *C. reinhardtii*. (**A**) – (**B**) Summary of *PHAS* loci that were induced (**A**) or repressed (**B**) in total siRNA generation by at least five-fold during phosphate starvation, sulfate starvation, or both.
